# *TGG1* and *TGG2* mutations impair allyl isothiocyanate-mediated stomatal closure in *Arabidopsis thaliana*

**DOI:** 10.1007/s00709-025-02039-z

**Published:** 2025-02-03

**Authors:** Kadri Oumaima, Mohammad Shakhawat Hossain, Wenxiu Ye, Eiji Okuma, Mohammad Issak, Mohammad Mahbub Islam, Misugi Uraji, Yoshimasa Nakamura, Izumi C. Mori, Shintaro Munemasa, Yoshiyuki Murata

**Affiliations:** 1 Graduate School of Environmental and Life Science, Okayama University, Okayama, 700-8530 Japan; 2 Institute of Advanced Agriculture Science, Peking University, Beijing, 100-871 China; 3 Department of Agricultural Botany, Sher-E-Bangla Agricultural University, Sher-E-Bangla Nagar, Dhaka, 1207 Bangladesh; 4 Institute of Plant Science and Resources, Okayama University, Kurashiki, Okayama 710–0046 Japan

**Keywords:** Allyl isothiocyante, *Arabidopsis thaliana*, Cytosolic free calcium, Myrosinase, Stomatal closure

## Abstract

Myrosinase, referred to as thioglucoside glucohydrolase (TGG), plays a crucial role in plant physiology through catalyzing the hydrolysis of glucosinolates into bioactive isothiocyanates. In *Arabidopsis thaliana*, the myrosinases TGG1 and TGG2 are essential for abscisic acid- and methyl jasmonate-induced stomata closure. Allyl isothiocyanate (AITC), one of myrosinase products, triggers stomatal closure in *A. thaliana*. We investigated stomatal responses to AITC to clarify the role of TGG1 and TGG2 in Arabidopsis guard-cell signaling. Allyl isothiocyanate at 50 μM and 100 μM induced stomatal closure in the *tgg1* and *tgg2* single mutants but not in the *tgg1 tgg2* double mutant. Furthermore, AITC at 50 μM induced the production of reactive oxygen species and nitric oxide, cytosolic alkalization, and oscillations in cytosolic free calcium concentration in guard cells of both wild-type and mutant plants. These findings suggest that TGG1 and TGG2 are involved in AITC signaling pathway through interaction with signal component(s) downstream of these signaling events, which is not accompanied by hydrolysis of glucosinolates because of the difference in subcellular localization between enzymes (myrosinases) and substrates (glucosinolates).

**Supplementary Information:**

The online version contains supplementary material available at 10.1007/s00709-025-02039-z.

## Introduction

Stomatal pores, bordered by pairs of specialized guard cells, are vital for regulating gas exchange and transpirational water loss. These guard cells are highly responsive to various biotic and abiotic signals, including plant hormones, light, drought, and pathogen attacks (Shimazaki et al. 2007; Murata et al. 2015; Ye et al. 2015).

Myrosinase (thioglucoside glucohydrolase (TGG), EC 3.2.1.147) can hydrolyze glucosinolates and is one of the most prominent proteins in cruciferous plants (Zhao et al. 2008; Islam et al. 2009). Isothiocyanates are most important products of glucosinolates in reaction to infliction by herbivores, insects, bacteria, and other stress factors (Bhat and Vyas 2019) and modulate stomatal movement triggered by light and phytohormones like abscisic acid (ABA) and methyl jasmonate (MeJA) (Zhao et al. 2008; Islam et al. 2009). In Arabidopsis, six myrosinase genes have been discovered, with TGG1 and TGG2 being enzymatically active (Barth and Jander 2006). Moreover, the *tgg* mutation decreased myrosinase activities in the rosette leaves in Arabidopsis based on the sinigrin assay and stomatal closure induced by ABA and MeJA was impaired in the *tgg* double mutant plants (Islam et al. 2009). Islam et al. (2009) concluded that TGG1 and TGG2 redundantly function downstream of ROS production and upstream of [Ca^2+^]_cyt_ in ABA and MeJA signaling pathways. Moreover, TGG1 deficiency decreased sensitivity to ABA and impaired response to wound-induced signals (Zhao et al. 2008).

Allyl isothiocyanate (AITC) is the hydrolysis product of the glucosinolate sinigrin and initiates stomatal closure in Arabidopsis (Khokon et al. 2011; Hossain et al. 2013; Murata et al. 2015). Furthermore, ITCs including AITC profoundly trigger stomatal closure in Arabidopsis (Afrin et al. 2020). Khokon et al. (2011) employed the 1,2-benzenedithiole-based cyclocondensation assay to show that TGG1 and TGG2 catalyse the hydrolysis reaction of glucosinolates for ITC production in the grinded leaves of Arabidopsis althought the cyclocondensation assay does not allow us to non-destructively measure ITCs in guard cells. Hence, if the myrosinases, TGG1 and TGG2, function as hydrolysis enzymes to produce ITCs in ABA and MeJA signaling, it can be expected that AITC induces stomatal closure in the *tgg1 tgg2* mutant because AITC is one of myrosinase reaction products. However, it may not be expected that ABA and MeJA promote the hydrolysis of glucosinolates in guard cells because myrosinases are spatially separated from their substrate glucosinolates in the plants (Bhat and Vyas 2019), that is, neither ABA nor MeJA is likely to induce the breakdown of glucosinolate mediated by TGG1 and TGG2 in Arabidopsis. To our knowledges, it remains to be clarified whether the myrosinases, TGG1 and TGG2, are involved in ITC-induced stomatal closure and whether AITC induces stomatal closure in the *tgg1 tgg2* mutants.

In this study, we examined stomatal responses of the *tgg* mutants to AITC in order to clarify whether TGG1 and TGG2 are involved in AITC-induced stomata closure and whether TGG1 and TGG2 have functions other than ITCs production in the guard-cell signaling pathways.

## Material and methods

*Arabidopsis thaliana* ecotype Columbia-0 (WT) and *tgg1* (SAIL_786_B08), *tgg2* (SALK_038730), and *tgg1 tgg2* mutant lines were grown in a controlled environment, genomic PCR was used to confirm the mutant lines, and for experimental use, 4- to 5-week-old rosette leaves were harvested.

Stomatal aperture was determined using the method outlined by Munemasa et al. (2019), three independent experiments were carried out (*n* = 3). Guard cells were exposed to 50 μM AITC to trigger the generation of ROS and NO, with ROS detected by 50 μM H_2_DCF-DA (Sigma) and NO detected by 10 μM DAF-2DA (Sigma) (Munemasa et al. 2007). Islam et al. 2010 protocol was used to monitor the guard cells cytosolic pH using BCECF-AM (Sigma) fluorescent dye. ImageJ software was utilized to analyze the images, and experiments were replicated three times. To visualize changes in cytosolic calcium concentration, the guard cells of Arabidopsis were loaded with the Ca^2+^ indicator YC3.6 (Hossain et al. 2011). The ratio of fluorescence intensity at (480/535 nm) was measured, and fluctuations greater than 0.1 in the ratio were counted as oscillations.

Analysis of variance (ANOVA) statistical analysis was employed using Tukey’s test to assess the significance of mean values differences (*p* < 0.05). The χ2 test was used to compare between WT and the *tgg* mutants in the elevation of [Ca^2+^]_cyt_ frequency induced by AITC. statistical significance was defined as *P* < 0.05. The genome initiative numbers for the genes of Arabidopsis mentioned in this article are: TGG1 (At5g26000) and TGG2 (At5g25980).

## Results

**Fig. 1:**
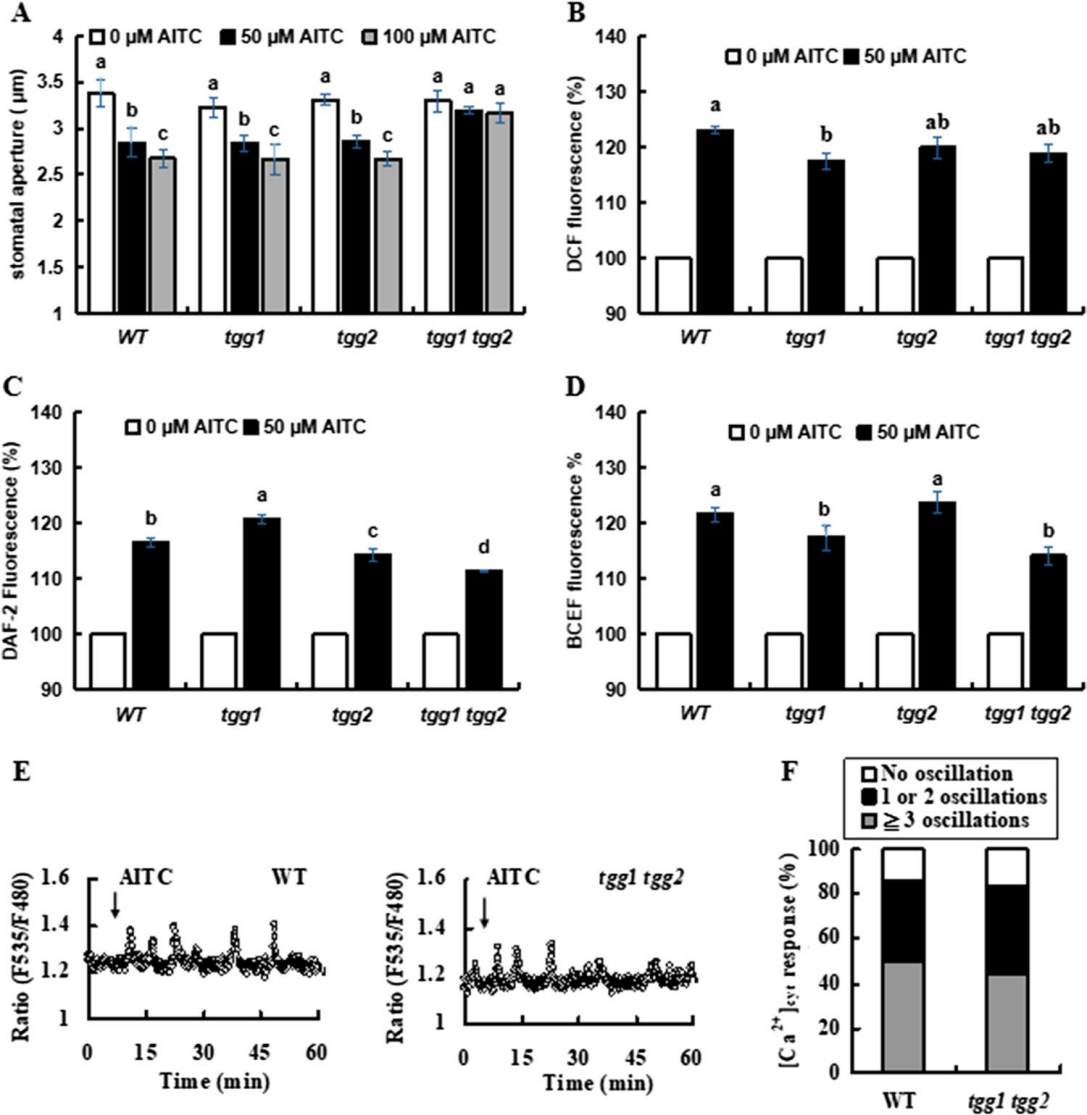
**A **Stomatal aperture in Arabidopsis in response to allyl isothiocyanates (AITC). **B **and **(C)** Production of ROS and NO induced by 50 μM AITC in guard cells of the wild-type and *tgg* mutants. **D **Cytosolic alkalization induced by 50 μM AITC in guard cells of the wild-type and *tgg* mutants. Data are presented as mean ± SE (*n* = 3, with > 60 stomata). Bars with the same letter are not significantly different at *P* < 0.05. **E **Representative [Ca^2+^]_cyt_ elevation (F_535 nm_/F_480 nm_) in 50 µM AITC-treated WT guard cells and *tgg1-3 tgg2-1* guard cells. **F **A percentage bar chart illustrating the frequency of AITC-induced [Ca.^2+^]_cyt_ oscillations in WT (*n* = 22) and *tgg1-3 tgg2-1* guard cells (*n* = 18)

The stomatal responses to exogenous AITC at 50 μM and 100 μM of wild type (WT) and *tgg1*, *tgg2*, and *tgg1 tgg2* mutants were examined. Treatments with AITC at 50 μM and 100 μM induced stomatal closure in WT (*p* < 0.05), *tgg1* mutant (*p* < 0.05), and *tgg2* mutant (*p* < 0.05) but not in the *tgg1 tgg2* mutant (*p* < 0.05) (Fig. 1A).

To make it clear how TGG1 and TGG2 participate in the stomatal closure, we assessed the accumulation of reactive oxygen species (ROS) and nitric oxide (NO) in the wild type and *tgg* mutants. Treatment with 50 μM AITC induced ROS and NO accumulation in the guard cells of *tgg1* (*p* < 0.05), *tgg2* (*p* < 0.05), and *tgg1 tgg2* (*p* < 0.05) mutants as well as in the guard cells of wild type (*p* < 0.05) (Fig. 1B, C). These results suggest that neither TGG1 nor TGG2 functions upstream of production of ROS and NO in AITC signaling in guard cells.

Cytosolic pH in guard cells of wild type and *tgg* mutants was monitored using BCECF-AM. Exogenous AITC at 50 μM induced cytosolic alkalization in the guard cells of *tgg1* (*p* < 0.05), *tgg2* (*p* < 0.05), *tgg1 tgg2* (*p* < 0.05) mutants as well as the WT plants (*p* < 0.05) (Fig. 1D). These results suggest that neither TGG1 nor TGG2 functions upstream of cytosolic alkalization in the AITC signaling in guard cells.

Elevation of cytosolic free calcium concentration ([Ca^2+^]_cyt_) in guard cells expressing a Ca^2+^-sensing fluorescent protein YC3.6 was observed. When WT guard cells were treated with 50 μM AITC, transient elevation in [Ca^2+^]_cyt_ was observed in 86% of the cells (n = 19 out of 22 cells; Fig. 1E, F). Meanwhile, [Ca^2+^]_cyt_ elevation was in 82% of the *tgg* double mutant treated with 50 μM AITC (n = 15 of 18 cells; Fig. 1E, F). There was no significant difference in the frequency of [Ca^2+^]_cyt_ elevation between WT and the double mutant (*P* = 0.789), suggesting that neither of the myrosinases functions upstream of [Ca^2+^]_cyt_ elevation in the AITC signaling in guard cells.

## Discussion

A hydrolysis product of sinigrin, AITC, triggers stomatal closure at physiological concentrations in one of glucosinolate-producing plants, *Arabidopsis thaliana* (Fig. 1A), which is in agreement with our previous results (Khokon et al. 2011; Hossain et al. 2013). Furthermore, AITC induces stomatal closure in *Vicia faba*, which does not contain myrosinases or glucosinolates (Sobahan et al. 2015). These results suggest that ITCs including AITC are inducers to trigger stomatal closure regardless of biosynthesis of glucosinolates.

ABA- and MeJA-induced ROS production is mediated by NADPH oxidases (Islam et al. 2010) while AITC-induced ROS depends on SHAM-sensitive apoplastic peroxidases (Hossain et al. 2013). The AITC-induced ROS production was not impaired in *tgg1 tgg2* mutant (Fig. 1B), similar to ABA- and MeJA-induced ROS in this mutant (Islam et al. 2009), indicating that TGG1 and TGG2 do not function upstream of ROS production in guard-cell AITC signaling. RCS-induced stomatal closure, cytosolic alkalization, and [Ca^2^⁺]_cyt_ elevation are disrupted in the *tgg1 tgg2* mutant while AITC-induced alkalization and [Ca^2^⁺]_cyt_ elevation in the double mutant are not impaired (Fig. 1D, E, F; Rhaman et al. 2020), suggesting RCS signaling is not totally overlapped with AITC signaling in guard cells. ABA and MeJA also induce [Ca^2^⁺]_cyt_ elevation in both WT and *tgg1 tgg2* (S1), confirming that TGG 1 and TGG2 are not upstream of calcium signaling. Notably, the mutation impairs ABA- and MeJA-induced stomatal closure but not Ca^2^⁺-induced closure (Islam et al. 2009; Rhaman et al. 2020), which can be attributed to differences in ROS production or upstream signals.

Myrosinase, TGG1, was outstandingly abundant guard-cell proteins and the *tgg1* mutation suppressed inhibition of plasma membrane inward-rectifying potassium channels by ABA (Zhao et al. 2008). Moreover, TGG1 and TGG2 were redundantly involved in ABA and MeJA signal transduction pathways in guard cells of *A. thaliana* (Islam et al. 2009). However, most of plants do not contain myrosinases or glucosinolates but can close stomata in response to ABA and MeJA, indicating that myrosinases are not a common signaling component essential to ABA- and MeJA-induced stomatal closure. In plants such as Arabidopsis and brassica family, myrosinases hydrolyze glucosinolates to form toxic ITCs as defensive substances when bacteria, fungi, insects and herbivores attack the plants (Barth and Jander 2006; Andersson et al. 2009). However, neither ABA nor MeJA is likely to induce the hydrolysis of glucosinolate mediated by TGG1 and TGG2 in Arabidopsis because of the difference in subcellular localization (Bhat and Vyas 2019). Therefore, TGG1 and TGG2 may interact directly with downstream signal component(s) such as ion channels that are related to stomatal closure.

## Conclusion

We can conclude that TGG1 and TGG2 play as a positive regulator downstream of production of ROS and NO, cytosolic alkalization, and [Ca^2+^]_cyt_ elevation, where TGG1 and TGG2 may directly interact with downstream signal component(s) such as ion channels to modulate their activities in Arabidopsis guard-cell signaling pathways.

## Supplementary Information

Below is the link to the electronic supplementary material.

Supplementary file1 (PDF 1499 KB)

## Data Availability

The analyzed data sets generated during the study are available from the corresponding author upon reasonable request.

## Abbreviations

ABA: abscisic acid
AITC: allyl isothiocyanate
BCECF-AM: 2ˊ,7ˊ-bis-(2-carboxyethyl)-5,(6)-carboxyfluorescein acetoxymethyl ester
[Ca^2+^]_cyt_: cytosolic free calcium concentration
DAF-2DA: 4,5-diaminofluorescein-2 diacetate
H_2_DCF-DA: 2ˊ,7ˊ-dichlorodihydrofluorescein diacetate
ITC: isothiocyanate
MeJA: methyl jasmonate
ROS: reactive oxygen species
TGG: thioglucoside glucohydrolases
WT: wild type
YC.3.6: Yellow Cameleon 3.6
