# Supplementary material for: TGG1 and TGG2 mutations impair allyl isothiocyanate-mediated stomatal closure in Arabidopsis thaliana
Source: Protoplasma. 2025 Feb 3;262(4):1023–7. doi: 10.1007/s00709-025-02039-z (PMC12141395; doi:10.1007/s00709-025-02039-z)
Supplement: Supplementary file 1 — Supplementary file1 (PDF 1499 KB) [file 709_2025_2039_MOESM1_ESM.pdf]

SHORT COMMUNICATION

Title: *TGG1* and *TGG2* Mutations Impair Allyl Isothiocyanate-Mediated Stomatal Closure in *Arabidopsis thaliana*

Author names and affiliations:

Kadri Oumaima[1], Mohammad Shakhawat Hossain[1], Wenxiu Ye[1,2], Eiji Okuma[1], Mohammad Issak[1,3], Mohammad Mahbub Islam[1,3], Misugi Uraji[1], Yoshimasa Nakamura[1], Izumi C. Mori[4], Shintaro Munemasa[1], and Yoshiyuki Murata[1,*].

[1]Graduate School of Environmental and Life Science, Okayama University, Okayama 700-8530, Japan

[2]Institute of Advanced Agriculture Science, Peking University, Beijing, 100-871, China.

[3]Department of Agricultural Botany, Sher-e-Bangla Agricultural University, Sher-e-Bangla Nagar, Dhaka 1207, Bangladesh

[4]Institute of Plant Science and Resources, Okayama University, Kurashiki, Okayama 710–0046, Japan

[*]Corresponding author. Tel.: +81-86-251-8310; fax: +81-86-251-8388.

e-mail address: muta@cc.okayama-u.ac.jp

Supplementary Information (S1)

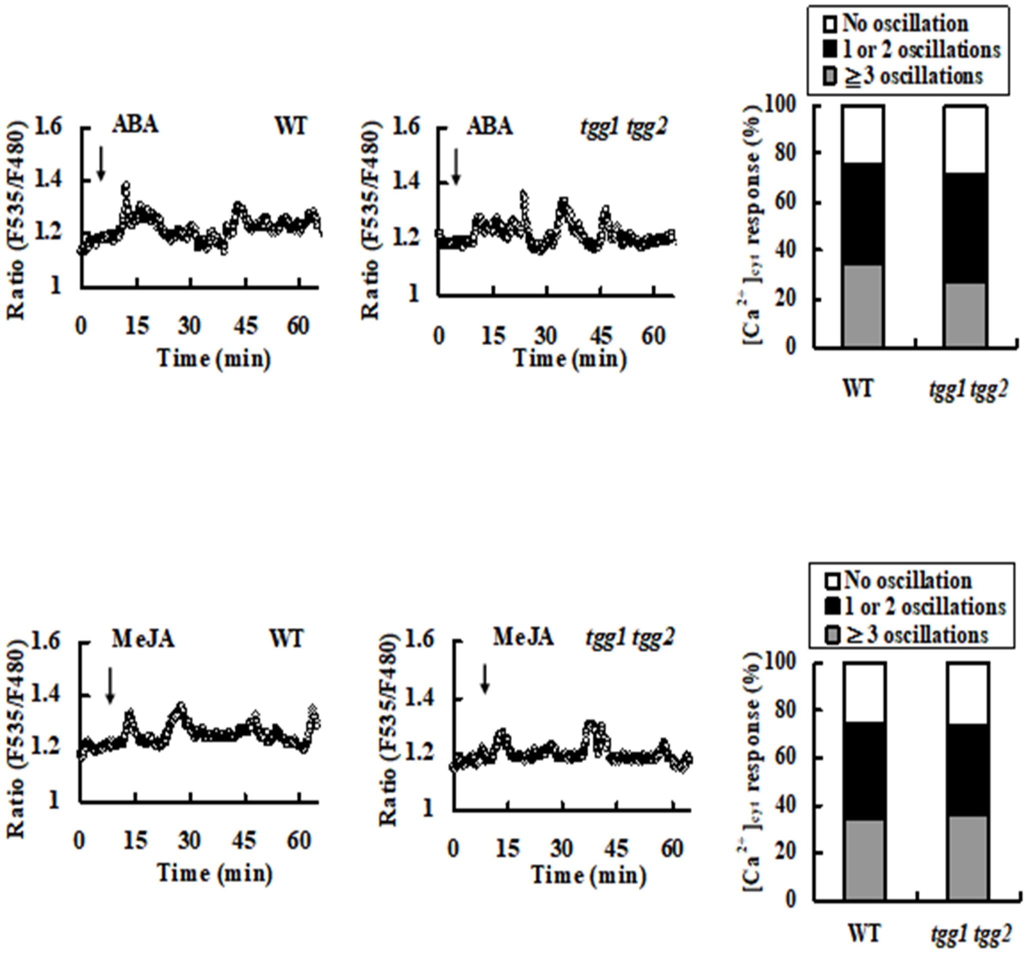

10 µM ABA- and MeJA-induced $[Ca^{2+}]$cyt oscillations in WT guard cells and tgg1-3 tgg2-1 guard cells. A percentage bar chart illustrates the frequency of ABA- and MeJA-induced $[Ca^{2+}]$cyt oscillations in WT ($n = 20$) and tgg1-3 tgg2-1 guard cells ($n = 19$). The arrow indicates when guard cells were treated with AITC, ABA or MeJA. Error bars represent SE.
